# Resignation of Professor Short

**Published:** 1849-07

**Authors:** 


### THE WESTERN JOURNAL.

THIRD SERIES.—VOL. IV.—NO. I.

LOUISVILLE, JULY 1, 1849.

RESIGNATION OF PROFESSOR SHORT.

Professor Short expressed early last autumn to some of his colleagues a wish, on account of enfeebled health, to retire from his duties in the Medical Department of the University of Louisville; but as it was believed that the failure of his health was only temporary, he was dissuaded from resigning at that time. His health, we are happy to state, has been entirely restored, but the wish to escape from the labors and anxieties of office has continued in full force, and has been, at length, carried out. His letter of resignation was sent to the Board of Trustees on the 18th of June. The Board, as a just compliment for his long and faithful services to the institution, elected him "*Emeritus* Professor of Materia Medica and Medical Botany."

Professor Short has retired from a lucrative and honorable station in the prime of life, with all his faculties of mind and body in full vigor, and will now, we trust, devote his leisure to the completion of a task which the profession has long expected at his hands. For many years, he has been engaged in collecting the materials for a work on the Botany of Kentucky. His herbarium is one of the most extensive and complete in the country, and few men in it are as familiar with its flora. He is capable of producing a work of great interest, which would confer credit upon the State, and be sought after by the scientific men of all countries.

The colleagues of Professor Short, on learning that his letter of resignation had been accepted by the Board, addressed to him the following communication:

"LOUISVILLE, June 19, 1849.

"*Dear Sir—*

It has been known to your colleagues for some time past, that it was your wish, at the first convenient moment, to relinquish the chair of Materia Medica and Medical Botany which you have so long held with credit to yourself and advantage to the school. It would have been most agreeable to your colleagues to have seen your connection with the school continued; but while such has been their feeling, they have not deemed that they had a right to urge you to a further sacrifice of your comfort and convenience. They are unwilling, however, that your official connection with them should be dissolved without an expression, on their part, of their sense of the important services you have rendered to the school, not only by your learned and able courses of instruction, but by your judicious counsels in the administration of its affairs. Nor can they allow the opportunity to pass without expressing to you the pleasure which they have derived from their official and personal association with you. They beg you to be assured that you carry with you into your retirement their highest regard and best wishes, and that they remain,

Very truly, your friends,

J. COBB,
S. D. GROSS,
H. MILLER,
L. P. YANDELL.

